# In our own eyes: ethical dilemmas and insights encountered by researchers conducting qualitative research in high ambient temperatures in Kilifi, Kenya

**DOI:** 10.1093/inthealth/ihad115

**Published:** 2023-12-26

**Authors:** A Lusambili, S Chabeda, P Khaemba

**Affiliations:** Environmental Health and Governance Centre, Leadership and Governance Hub, School of Business, Africa International University, Kenya; Institute for Human Development, Aga Khan University, East Africa; Institute for Human Development, Aga Khan University, East Africa; Institute for Human Development, Aga Khan University, East Africa

**Keywords:** challenges, ethics, high ambient temperatures, methods, pregnant and post-partum

## Abstract

We reflect on our fieldwork experience from the Climate Heat Maternal and Neonatal Health Africa (CHAMNHA) project in Kilifi, Kenya, which focused on studying the effects of extreme heat on women during pregnancy, delivery and the post-partum period. We describe the ethical and practical challenges encountered, highlighting valuable lessons learned. We propose potential solutions to address issues concerning the reciprocity of vulnerable participants and the provision of childcare and food for accompanying children. Further, we address challenges related to engaging specific participants, interview cancellations attributed to extreme temperatures and discuss the perpetuation of inequalities by ethics and academic institutions. With the anticipated increase in research at the intersection of climate change–induced heat exposure and its impacts on human populations, research institutions and ethics committees in low- and middle-income countries are responsible for instituting guidelines that account for the risks for the subjects under study and the field researchers.

## Background

The Climate Heat Maternal and Neonatal Health in Africa (CHAMNHA) project conducted ethnographic research in Kenya and Burkina Faso to understand the effects of extreme heat exposure on pregnant and post-partum women. In Kenya, the fieldwork was conducted in rural Kaloleni and Rabai subcounties in 2022. The interviewed participants lived in sparsely distributed communities connected by rough road networks, with temperatures ranging from 25°C to 35°C. Families were typically large, with households consisting of 5 to 12 members, organised along traditional lines, with grandparents, daughters-in-law, children and extended family members residing in a single household. Due to the area's remote nature, health facility staff and community health volunteers, with guidance from researchers, recruited participants (including pregnant and post-partum women, male spouses and mothers-in-law) who were interviewed at the nearest health facility.

Our research was undertaken during the peak of the hottest months, and researchers faced several challenges. The lack of research on the intricacies of conducting fieldwork amidst elevated temperatures in rural coastal and other East African locales became apparent. We present this reflection article to share our experiences and provide potential solutions to future researchers regarding the ethical, social, cultural and environmental challenges associated with ethnographic research on environmental hazards. By sharing insights gained from our fieldwork, we hope to contribute valuable lessons for future researchers grappling with the distinctive demands of ethnography in high-temperature settings. We aim to foster an understanding of the ethical considerations, social dynamics, cultural nuances and environmental complexities inherent in the pursuit of knowledge in such challenging conditions.

## Ethical challenges

### Participant's reciprocity and accompanying children

Qualitative researchers expect to encounter methodological and ethical challenges,^1^ including issues related to unresolved participant reciprocity, fair research conduct and physical or environmental hazards. These challenges become particularly salient when researching marginalized communities in rural settings in low- and middle-income countries (LMICs) and when academic institutions lack a comprehensive understanding of research ethics governing participant interactions. Some educational research institutions in LMICs may develop field research policies without considering research ethics and their implications for participants. Consequently, field researchers might refrain from reporting ethical dilemmas due to the lack of good institutional practice and research governance. Due to financial constraints, researchers may also prioritise the pursuit of research questions over the well-being of participants. Unforeseen problems can emerge while conducting climate change–related research in rural settings like Kilifi, known for experiencing high temperatures.

High temperatures raise several challenges for participants. These include asking women to walk long distances in the heat to attend interviews when transportation expenses should have been prearranged and overlooking the budget for childcare and children's refreshments in advance. Problems occurred because mobile cash transfers could not be completed to support transportation costs among rural residents before or immediately after the interviews.

When researchers arrived at the interview venue, it was 31°C, and Karima was waiting to be interviewed.… She had with her two other children below age 5 who were hungry and exhausted. She had trekked in the heat for 45 minutes to get to the interview venue. After the interview, it was 5 PM, and Karima was expecting to be reimbursed $3 in cash for her transport costs to enable her to hire a motorbike to return home. Researchers could not help because the academic research host institutional guidelines discourage reimbursing participants’ costs in cash but instead recommend direct transfer to her phone. Although these processes had been explained to Karima during recruitment, Karima still expected cash on this day. She became tearful as she had budgeted to buy weekly food from $3. She had no other cash on her…and walking back home for 45 minutes with three children after 5 PM was risky.

Karima's story mirrors the many similar experiences of mothers in this study. After trekking for almost an hour, often accompanied by their children, they were left feeling exhausted, restless and hungry. They had anticipated that researchers would provide food for their children and reimburse their transport costs in cash.

Additionally, study participants were expected to complete forms and provide their phone numbers to be reimbursed for their expenses. However, not all potential interviewees were literate, had mobile phones or were proficient in mobile money transfers. The majority of our female research participants did not own mobile phones. When advised by researchers to provide the mobile phone numbers of their spouses or other household members, they hesitated, fearing the money might not reach them. This issue was especially evident in focus group discussions with older women, most of whom did not own a phone. The negotiation process and the challenge of finding community members with phones that participants trusted proved time-consuming for researchers and interviewees.

In Kilifi, women bear the primary responsibility for caregiving duties, meaning that mothers who cannot afford childcare often travel with their children. The researchers involved in this study did not have a childcare plan in place to address this problem. Consequently, young children had to be present during discussions, and researchers had to provide them with refreshments initially intended for other interview participants, using their own funds to purchase additional refreshments.

#### Reflections and potential solutions

These dilemmas underscore the importance of future research budgets accounting for appropriate means of payment and also budgeting for childcare and refreshments for family members accompanying participants. Our experiences also highlight the necessity for ethics review committees in LMICs to ensure that studies involving mothers and young grandparents, who often serve as caregivers, factor in childcare expenses in their budgeting.

University institutional guidelines prohibit non-staff members from being transported in university cars. On many occasions, we drove back from the interview locations by car without being able to assist vulnerable women who had invested their time in our research by providing transportation back to their homes. This situation felt unethical and inhumane, as it assumed that participants could easily access planned research venues and were not encumbered by other daily responsibilities.

University institutions also do not reimburse staff for out-of-pocket expenses, such as helping participants with transportation costs. Therefore, the policy unfairly burdened researchers and exacerbated inequalities. Research institutions adopting a similar approach must consider fairer methods, such as providing cash in advance to accommodate vulnerable participants during the interview process to avoid lengthy waiting periods.

Reflexivity has the potential to address methodological obstacles, enhance participant autonomy and capacity and contribute to the possibility of conducting rigorous and valid research by promoting ethical practices and methodological validity.^2^ Participation should always remain voluntary and free from coercion and should offer positive and beneficial experiences to participants while minimizing potential risks.^3,4^ To maintain participant safety, researchers must frequently confront ethical and contextual methodological issues that may arise during the research process. Based on our experiences, we urge all Kenyan and LMIC academic institutions with these detrimental policies to rectify them. We strongly emphasise to researchers operating in similar settings the importance of diligently observing, identifying and responding to emerging ethical, methodological and field challenges throughout the research process and advocating for necessary adjustments within their institutions.

The intricate aspects of ethical considerations suggest that research practices should distinguish between procedural ethics (formal processes involving approval from research ethics committees) and practical ethics (involving researchers responding to emerging issues during implementation).^5^ While consensus-based approaches are used to obtain legal consent, researchers need to recognize and address context-dependent conditions and ethical contingencies that may arise during actual research practices.

Situations involving emerging ethical considerations can potentially disrupt planned research procedures unless they are carefully reflected upon and addressed. This underscores the need for researchers to reflect on the ethical and methodological complexities that may require a response tailored to the specific research context. Additionally, researchers should observe and adhere to the ethical procedures outlined in their research ethics applications and apply these principles in their research practices. Achieving this goal necessitates ethical reflexivity, calling upon researchers to adopt a flexible, ongoing and context-dependent process of critical scrutiny, interpretation and action concerning themselves as researchers and guided by the current situation.^6^

### Too hot to attend scheduled interviews

Our fieldwork was conducted during the hot season, as our focus was explicitly on heat-related health issues. However, this decision caused challenges, such as the need to cancel interviews and focus group discussions in the afternoon when it was too hot. Many participants failed to attend meetings, especially in the afternoons when temperatures peaked. This had several consequences, including rescheduling discussions, which extended the data collection period and affected project costs. Researchers incurred additional expenses in organizing refreshments for participants.

#### Potential solutions

As research in hot areas continues to gain prominence, it will require pre-planning and data collection in the early morning hours. This study demonstrates the necessity of striking a balance between risks and benefits for research participants. Assessing the risks and benefits associated with each research project is essential to ensure fairness and eliminate power imbalances. Emerging insights from the CHAMNHA qualitative study revealed the importance of finding an equilibrium between conventional research ethics requirements and contextual methodological considerations, particularly regarding the convenience of research participants during access to and from interview locations when it is hot to walk in the heat. While it may be more practical for field researchers to conduct interviews at participants’ homes, especially in settings with high temperatures, this approach may entail additional costs due to the dispersed nature of the homes, resulting in longer travel times. Future research in similar areas should consider allocating more time and resources for fieldwork to cover time lost for cancellations when it is hot.

### Hard to engage pregnant women

A challenge in researching pregnant women in traditionally patriarchal societies is establishing trust when discussing sensitive topics. They may be concerned about why they are being studied during this delicate time of pregnancy. Researchers are often considered part of the privileged group, which may make some vulnerable groups hesitant to trust them. During fieldwork, some post-partum and pregnant women were reluctant to divulge much information about their experiences of heat stress. Some informed us about social and cultural perceptions of pregnancy, where mothers are encouraged not to talk about unborn children, as it is believed to bring bad omens. We struggled to obtain valuable information from pregnant women individually. In contrast, post-partum and breastfeeding women cheerfully narrated their experiences of heat exposure during their pregnancy journey.

#### Reflections and potential solutions

Our experiences suggest that future research involving pregnant women in similar situations discussing unborn children should be planned carefully and, if possible, local healthcare workers should be used to gather information. This approach may help women feel more confident discussing pregnancy after a successful delivery. The CHAMNHA field experiences in Kilifi taught us various lessons, including the need to involve community members in the planning process. This utilises the community's knowledge about facilitating or hindering factors for successful research project implementation, such as people's behavioural characteristics during high temperatures. This helps in planning and scheduling interviews to avoid cancellations or interviewing participants under temperature stress.

## Conclusions

Research projects must uphold the highest ethical standards. They should clearly outline how ethical considerations will be addressed, elucidate potential ethical concerns, establish best research practices, foster positive relationships among researchers and other stakeholders and fulfil research obligations to society. For future studies conducted in similar settings, early planning is essential. Institutions in LMICs must commit to implementing measures that can reduce inequalities in the field.

## Data Availability

This is a reflective text and data is not available.
